# Non-invasive monitoring of hydraulic surge propagation in a wounded tobacco plant

**DOI:** 10.1186/s13007-018-0307-6

**Published:** 2018-05-25

**Authors:** Vladimíra Nožková, Petr Šmíd, Pavel Horváth, Miroslav Hrabovský, Petr Ilík

**Affiliations:** 1 Centre of the Region Haná for Biotechnological and Agricultural Research, Department of Biophysics, Faculty of Science, Palacký University, Šlechtitelů 27, 783 71 Olomouc, Czech Republic; 2 Institute of Physics of the Czech Academy of Sciences, Joint Laboratory of Optics of Palacky University and Institute of Physics AS CR, 17. listopadu 50a, 772 07 Olomouc, Czech Republic; 3 Regional Centre of Advanced Technologies and Materials, Joint Laboratory of Optics of Palacký University and Institute of Physics AS CR, Faculty of Science, Palacký University, 17. listopadu 12, 771 46 Olomouc, Czech Republic

**Keywords:** Hydraulic surge, Light diffraction, Local burning, Stem deformation, Tobacco

## Abstract

**Background:**

When a plant is wounded, a rapid hydraulic surge, acting probably as a systemic signal, spreads from the site of injury throughout the plant and leads to small transient deformation of tissues. So far, the propagation of hydraulic surge has been monitored by contact and thus potentially invasive methods.

**Results:**

Here we present a non-invasive optical method, which allows simultaneous monitoring of micrometric shift of two opposite stem margins. The usefulness of this method was demonstrated by the measurement of the hydraulic surge propagation in a tobacco (*Nicotiana tabacum* (L.) cv*. Samsun*) after burning of its upper leaf. We have observed transient narrowing the stem below the burned leaf, which started within a few minutes after local burning. The comparison of the shift of the stem margin following vascular trace of the burned leaf and the margin on the opposite side of the stem has revealed that the stem deformation is highly asymmetric.

**Conclusions:**

This optical method represents a novel tool to investigate the mechanism of systemic response of plants to local damage. Our results points out the complexity of the relationship between hydraulic surge propagation and stem deformation.

**Electronic supplementary material:**

The online version of this article (10.1186/s13007-018-0307-6) contains supplementary material, which is available to authorized users.

## Background

During their lifetime, plants in nature are often exposed to damaging factors leading to their wounding. To be able to survive, they have to respond promptly to the stress factors not only at the wounded site (local reaction), but also in distant unwounded tissues (systemic reaction). This systemic reaction is dependent on a moving signal, which informs remote unwounded tissue about the external disturbance. The identification of this signal is still in process, because the systemic defense response involves a complex network of signaling components. One of the fastest stress signals in plants could be a hydraulic surge. It spreads from the wounded plant area to distant unwounded tissues, where it could evoke plant defense responses [e.g. 1–6]. The propagating hydraulic surge causes a small deformation of plant tissues [e.g. 1, 7], which originates probably in the changes in turgor of affected cells or in the changes of hydrostatic pressure in xylem vessels. Thus, the hydraulic surge propagation in a plant can be monitored via the measurement of plant tissue deformation.

The measurement of the deformation of plant tissue is usually realized by contact methods. For example, Mancuso [7] measured stem deformation of grapevine after local burning of one leaf by using four active strain gauges placed on the opposite sides of the shoot and connected in so-called Wheatstone bridge. The author interpreted his results as an initial small stem narrowing followed by a pronounced increase in stem diameter. However, it is important to note that this technique gives credible results only when two of the four strain gauges are taken as reference ones (i.e. when these gauges detect only negligible deformations during the measurement). This condition is not generally fulfilled during the measurement of potentially complex stem deformations. If we assume that the responses of individual gauges are independent, it is impossible to judge on the type and magnitude of transversal stem deformation. Another approach to detect stem deformations after burning of an upper leaf was published by Stanković et al. [8]. These authors used angular position-sensing transducers that were placed vertically and horizontally on a sunflower stem (attached by a glue droplet) in order to measure changes in the stem length and diameter, respectively. After burning, the authors have observed stem elongation accompanied by stem widening, indicating a small increase in stem volume. This change was followed by slow return to the initial state and subsequent narrowing of the stem. A similar method, based on the measurement of deformation by linear displacement transducers, was used by Malone [1] for the monitoring of changes in leaf thickness after local burning of the neighboring leaf in wheat seedlings. Using this method an initial increase in leaf thickness was observed, followed by a slow return to the initial state.

All the above discussed methods require direct physical contact of a sensor with the measured plant area, which can affect/disturb plant tissue and thus can also distort the results. These considerations led us to an idea to measure the stem deformations induced by the propagating hydraulic surge by a non-invasive optical method without any contact with the measured plant area. Although optical techniques are more technical- and skill-demanding, they are more sophisticated and their non-invasiveness and high sensitivity supersede the conventional contact methods. Theoretically, the most suitable optical method for this purpose would be holographic interferometry, as it can determine a position change in fractions of a light wavelength. Even though this technique has been used several times in the 1980s, e.g. for the measurement of plant elongation [e.g. 9], the practical use of this technique for the detection of stem deformation is very limited. The limitation is caused by intensive static and dynamic light scattering on a plant surface. While the former is caused by the roughness of a plant surface, the latter is associated with the movement of scatters such as cell organelles [e.g. 10]. These scatterings significantly affect the resulting pattern and complicate the subsequent analysis of the pattern changes due to deformations.

**Fig. 1:**
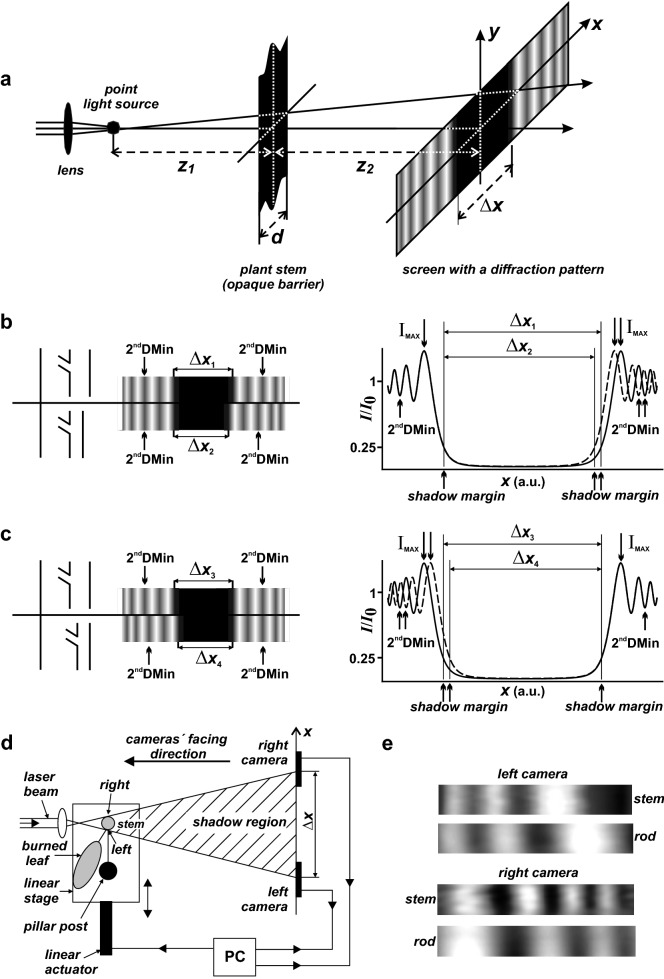
Principle of the measurement technique. **a** Divergent light rays diffract at margins of an opaque barrier (plant stem) and form a Fresnel diffraction pattern on a screen behind the stem. **b**, **c** Mathematical simulation of the shift of the stem margin and the corresponding diffraction pattern (a.u.—arbitrary units). **d** A scheme of the optical set-up. Right and left camera recorded the Fresnel diffraction pattern originating at right and left stem margins respectively. The measured tobacco plant was fixed to a pillar post and placed on a movable table driven by a linear actuator, which was connected to a PC. **e** Measured diffraction patterns from stem and steel rod margins

Here we present an alternative optical method, which is simpler and suitable for the monitoring of stem deformations. It is based on the measurement of the shift of the stem geometric shadow on the screen located behind the stem. The straight shadow margins on the screen, corresponding to stem margins, are affected by a characteristic light diffraction on the illuminated stem margins. When the screen is relatively close to the barrier (stem), we can see a typical near-field (Fresnel) diffraction pattern at both margins. The shape and other characteristics (e.g. brightness) of the diffraction pattern depend on the light wavelength, stem diameter and the distance between the stem and the screen. When the stem margins are shifted due to stem deformation, we can observe the corresponding shifts of the diffraction patterns (see Fig. 1).

In this work we describe an optical method for the monitoring of hydraulic surge propagation in the stem. The method employs Fresnel light diffraction on the stem margins to monitor stem deformation evoked by the hydraulic surge. Apart from the non-invasiveness, the method enables to detect an asymmetry in stem deformation.

## Methods

### Principle of the method and experimental set-up

A principle of the optical method for the measurement of stem deformation is demonstrated in Fig. 1a. A laser beam (He–Ne laser, *λ *=632.8 nm, 0.4 mW), focused by a lens to a point (point light source) in front of the stem, forms a spherical spot on the stem. A diameter of the spot (~ 12 mm) exceeds the diameter of the stem *d* (5–7 mm). The incident divergent light rays are diffracted at the stem margins (an opaque barrier) and form a typical diffraction pattern on a screen (Fig. 1a) [e.g. 11]. By varying the distances between the stem and the point light source (*z*_1_) and between the stem and the screen (*z*_2_), we can adjust the position and the shape of the fringes in the diffraction pattern. Determination of the distances *z*_1_ (8 mm) and *z*_2_ (297 mm) was based on our previous analysis [12] where the diffraction pattern showed undisturbed dumped swept-frequency sine behavior (nearly sinusoidal), which could be used for an easy location of bright and dark fringes. Figure 1b, c shows a theoretical profile of relative light intensity *I*/*I*_0_ on the screen along the *x*-axis, where *I*_0_ value represents the light intensity that would be detected without the opaque barrier. Figure 1b, c also shows a theoretical shift of the diffraction pattern evoked by a shift of the opaque barrier (stem margin) to the right and left. The width of the geometrical shadow region on the screen *∆x* is equal to *d*(*z*_1_ + *z*_2_)/*z*_1_; it increases with increasing stem diameter, increasing distance *z*_2_ and decreasing distance *z*_1_ and, according to theory [13], its borders are at the positions at which the *I*/*I*_0_ = 1/4. The borders are indicated by arrows (shadow margin) in Fig. 1b, c together with the maximum intensity of light (*I*_*MAX*_), which is not located at the margin of the geometrical shadow, but some distance away. Then the light intensity oscillates and the period becomes lower with increasing distance from the shadow margins (Fig. 1b, c).

A shift of the diffraction pattern corresponding to the shift of the stem margins was recorded by a pair of black and white cameras placed in the plane (*x*, *y*) at the distance *z*_2_ from the stem (Fig. 1a, d). The cameras were located on a horizontal optical rail running along the *x*-axis. The rail was perpendicular to the direction of the laser beam and at a position that allows the central row of the cameras to be approximately in the *xz*-plane running through the centre of the beam. The rail allowed the cameras’ location apart from each other by about width Δ*x* of the geometrical shadow. For example, the width of the geometrical shadow is Δ*x* = 229 mm for the stem of width *d* = 6 mm according to [12]. Each camera monitored a diffraction pattern at the corresponding margin of the barrier (stem). The vertical direction of fringes in the diffraction patterns confirmed that the incident laser beam falls perpendicularly on the stem. The cameras had a pixel size of 5.2 × 5.2 μm and 8-bit depth. Recorded images had a size of 1024 × 128 pixels (width × height) (Fig. 1e). When the margin of the barrier (stem) changes its position by ∆*a*, the corresponding set of bright and dark fringes moves by *C* ∆*a* in the plane (*x*, *y*) in the same direction. The *C* = (*z*_1_ + *z*_2_)/*z*_1_ defines the sensitivity of the optical set-up and is derived from the width of the shadow region (∆*x*). The light sensitive pixels in both cameras are spaced equally by ∆*p*. Provided that *N*∆*p* is a minimal differentiable change in the fringe position, where *N* is a number of pixels, then *C*/(*N*∆*p*) is a resolving power of the optical set-up. Our optimized set-up enabled a detection of a shift of a stem margin by about 1 μm. Optimal distances *z*_1_ and *z*_2_ in relation to the sensitivity *C* were established by our analysis [12]. That allowed the optical set-up to record the required minimum shift of stem margins. Table 1 summarizes distances and values valid for the presented experimental set-up.

**Table 1:** Summary of distances and values valid for the experimental set-up

Quantity	Value	Description
*z*_1_	8 mm	Distance between the stem and the point light source [12]
*z*_2_	297 mm	Distance between the stem and the screen [12]
Δ*x*	229 mm (for the stem width = 6 mm)	Width of the geometrical shadow [12]
Δ*p*	5.2 μm	Camera pixel size
*N*	7 pixels	Response of a fringe position to the stem margin shift by 1 μm
*C*	38.125	Sensitivity of the optical set-up

It should be noted that the set-up sensitivity *C* depends on distance *z*_1_ and *z*_2_. A shift of the stem by 0.1 mm to the cameras would slightly decrease the sensitivity (by 0.47), which would result in a small shift of the diffraction pattern (by 9% of the pixel width in case of the stem margin shift by 1 μm). Thus a stem shift in this range would influence the measurement insignificantly.

A shift of the diffraction pattern is evaluated as a shift of the second dark fringe, i.e. a second diffraction minimum (2^nd^DMin) in the diffraction pattern (see arrows in Fig. 1b, c), counted from the corresponding margin of the shadow region. We selected this fringe because it has the most convenient position and shape in the diffraction pattern compared to other fringes. Primarily, it is close enough to the margin of the shadow region and simultaneously it is properly narrow (see Fig. 1b, c), both factors significantly contribute to its precise location compared to other fringes. In addition, due to its narrow shape and middle position in the diffraction pattern, the second diffraction minimum can be easily placed in the center of the dimension-limited sensor of camera to leave enough space for its movement during our long-lasting measurement. A position of this second dark fringe was determined using custom-authored software (see Additional file 1). All recorded diffraction patterns were evaluated from the same line (row) of the camera image, which—in our optimized set-up—corresponded to a stem height of 0.14 μm.

### Preparation of a plant and calibration measurement

To prepare the plant for the measurement, we had to remove trichomes from the tobacco stem as they disturb the diffraction pattern produced by the stem margins. The trichomes were removed by gently rubbing a finger over the measured stem area and then the plant was left to rest for 3 days before the measurement to eliminate any possible effect of this procedure on the turgor of plant cells. After this procedure, the diffraction at the stem was comparable to the diffraction at a steel rod (Fig. 1e) [12] indicating that the stem can be considered to be an opaque barrier. We need to use an opaque barrier in our experiments, because in case of a partially transparent barrier, disturbances in diffraction pattern arise (see Additional file 2) resulting from the interference of light transmitted through the barrier and that diffracted by its edges. The disturbances would negatively affect determination of the required fringe position.

**Fig. 2:**
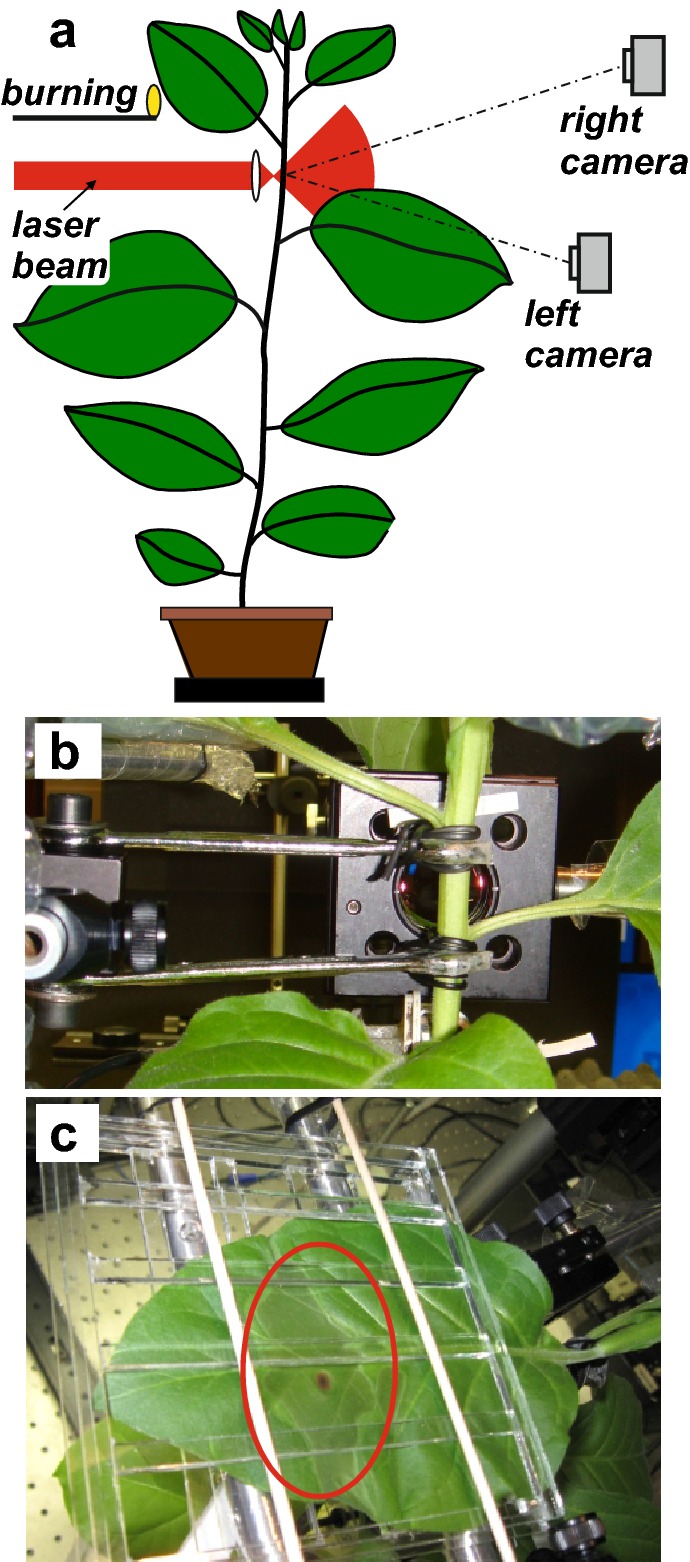
**a** A schematic experimental arrangement. **b** The tobacco stem fixed by two holders located above and below the measured stem area. **c** The burned leaf fixed in a glass holder. The final heat-treated area with a central burned spot is marked with a red oval

Our experimental set-up (Fig. 2a) required a fixation of the stem to prevent its spontaneous movement. The stem was fixed by two holders located above and below the measured stem area (Fig. 2b). The holders were fastened to a stable pillar post (Figs. 1d, 2b), which was placed together with the measured plant on a special movable table (linear stage equipped with an EncoderDriver linear actuator, Coherent, USA). The table was driven by a PC. The actuator enabled a precise shift of the table with a resolution of 0.02 μm. The whole set-up was placed on a rigid tabletop with broadband damping of vibrations (StableTop 250, MellesGriot, USA).

**Fig. 3:**
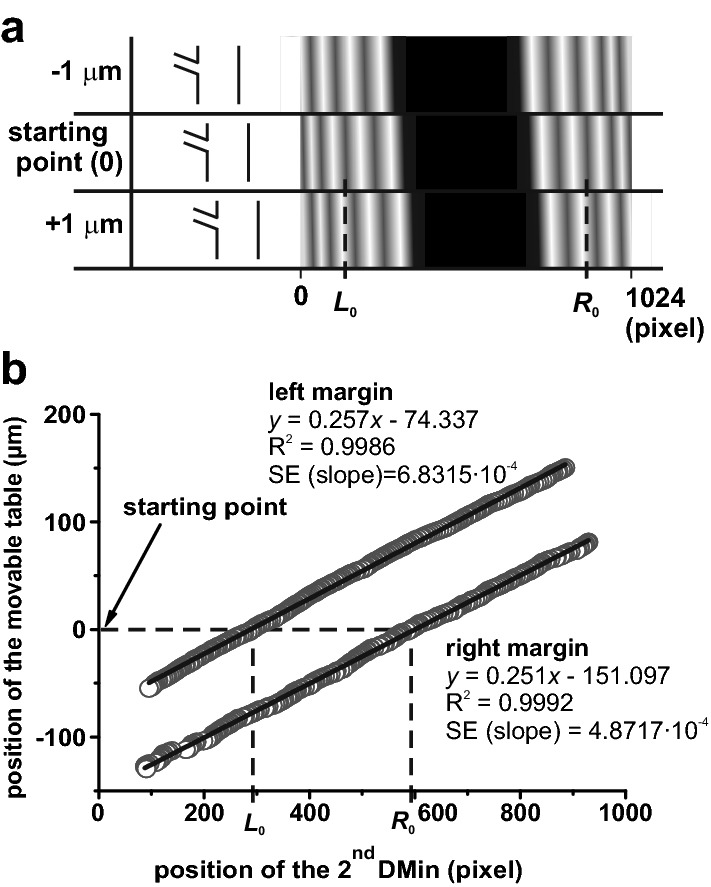
**a** A mathematical model of the shift of diffraction patterns due to the shift of a barrier (stem) by 1 μm to the left and to the right. **b** A calibration measurement. The tobacco plant was placed on a movable table and was shifted with a 1 μm step. The positions of the second dark fringes, counted from the corresponding margins of the shadow region, were determined in every stem position. The symbols *L*_0_ and *R*_0_ indicate coordinates of the second dark fringes corresponding to the starting position of the stem labeled as “0”. The relationship between the stem position and the coordinates of the second dark fringes was used for the calculation of calibration lines used for the evaluation of experimental data. SE means standard error

Before the measurement of the stem deformation, we performed a calibration measurement with a plant on the movable table. The purpose of the calibration is to determine the relation between the changes in 2^nd^DMin and physical stem deformations. We moved the table with a plant right and left by 150 μm with the 1-μm step (along the *x*-axis, see Fig. 1d) and recorded the corresponding diffraction patterns of the plant stem using the cameras. A simulation of the shift of the diffraction pattern is displayed in Fig. 3a. After recording of the diffraction patterns in all 301 positions along the trajectory of the moving table, we have evaluated the positions of the second diffraction minimum (see above) in the patterns and plotted the table shift as a function of the minimum positions (Fig. 3b). Then we used linear regression to obtain calibration lines, which were used for the calculation of stem deformation in the following experiment. Since the extent of the shift of the second diffraction minimum was larger than the detection width of the used camera sensor, the lines in Fig. 3b cover a smaller range of the table shift. Nevertheless, such a range was sufficient for the measurement.

### Measurement of stem deformation after burning

At first, we had to verify the stability of the fixed stem. The cameras recorded diffraction patterns from both stem margins with a frequency of 1 frame per second (fps) over a period of about 30 min. This period appeared to be long enough for the stabilization of the stem, i.e. its movement was negligible in comparison to its movement after burning. After this initial period, a leaf above the fixed part of the stem was burned (see below) and the cameras recorded the diffraction patterns with a higher frequency (3 fps) over a period of 10 min. Then the frequency was again lowered to 1 fps and the measurement continued for 135 min.

**Fig. 4:**
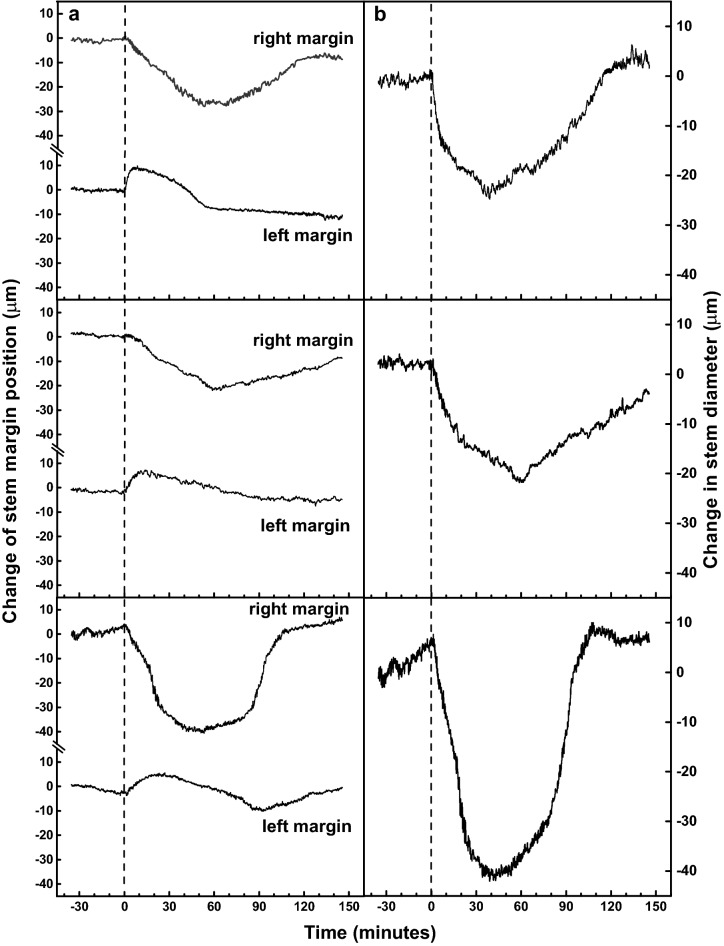
**a** Representative shifts of right and left margins of a tobacco plant stem induced by a local burning of the upper leaf (dashed line). **b** Estimated change in the stem diameter calculated as a difference between the shifts presented in **a**. Data from three individual plants are shown

After the experiment, we determined the coordinates of the second diffraction minimum of the pattern in the selected line of the camera sensor using the custom-authored software. The relative changes in the positions of right and left stem margin were calculated using the calibration lines (Fig. 3b). Finally, the detected positions of the stem margins of a measured plant were plotted as a function of time (Fig. 4).

### Plant material and burning process

*Nicotiana tabacum* (L.) cv*. Samsun* plants were cultivated in a growth chamber at 25 °C, RH 60%, under 16 h light (100 µmol photons m^−2^ s^−1^, 400–700 nm)/8 h dark cycles. Plants were grown in pots filled with seed soil substrate and were fertilized by KRISTALON solution (Hydro Agri Rotterdam, Netherlands) every week. Measurements were performed with tobacco plants that were 81–85 days old, 45–55 cm tall with 13–17 fully developed leaves.

A middle part of the upper half of the 12th fully developed leaf (numbered acropetally, the first fully developed leaf from plant top) was burned by a flame (Fig. 2a, c, see movie in Additional file 3). Before burning, the chosen leaf was fixed into a home-made glass holder to avoid any leaf movement during the burning process (Fig. 2c). To achieve well defined and reproducible burning of the leaf, a Teflon spoon was mounted to a stable stand in the close vicinity of the leaf. A small amount of ethanol (45 µl) was added to the spoon and ignited by a burning wooden stick. The burning process was started by a rotation of the firing spoon directly below the burned leaf and lasted 15 s. An additional movie file shows this in more detail (see Additional file 3). The distance between the spoon and the abaxial side of the burned leaf was about 20 mm and the temperature of the flame was 600–700 °C.

## Results and discussion

We have developed an optical method for the monitoring of hydraulic surge propagation in plants and applied it to the measurement in wounded tobacco plants. The hydraulic surge was initiated by a local burning of the 12th leaf. The section of the stem selected for the deformation measurement was located directly below the burned leaf (Fig. 2a, b). The distance between the measured area of the stem and the burned leaf area varied between 120 and 160 mm. We monitored a shift of two opposite margins before, during and after the burning. The plant was oriented in such a way that the left margin corresponded to stem margin directly below the burned leaf, i.e. on the leaf trace, and the right margin was on the opposite side (for other details see “Methods”).

Figure 4a shows representative deformation curves of the stem margins induced by the local burning. Typically, shifts of both stem margins started within a few minutes after the burning. Maximal shift of the stem margin has been usually reached much earlier on the left side of the stem (below the burned leaf; 7–20 min) than on the right side (about 1 h; Fig. 4a). However, the average amplitude of the shift of the right margin was much more pronounced (− 32 µm ± 7 µm on the right margin; + 9 µm ± 1 µm on the left margin; n = 3). After reaching the maximal shifts, the margins began to move back to their original positions. Figure 4b shows the changes in stem diameter calculated from the curves in Fig. 4a. Interestingly, we did not observe any trace of stem expansion immediately after the local burning, although such response has been frequently observed in leaves or stem after local burning using the contact techniques [e.g. 1, 2, 4, 8].

The phenomenon of stem contraction (narrowing) after local wounding can be interpreted by two hypotheses—a water potential gradient or osmotic processes evoked by sap flow in xylem. Taken into account the traditional plant physiology approach, during the burning process, many cells are damaged and the cuticle of the burned leaf is lost, resulting in immediate increase in transpiration and water loss from the burned leaf (see water condensation on glass holder of leaf during burning process, Additional file 3). This process is magnified by dry air surrounding the burned leaf. As a result, a rapid turgor decrease was visible in cells directly burned and in surrounding tissues, which were markedly flaccid (Fig. 2c). This indicates a rapid decrease in water potential of cells surrounding the burning site. Because a gradient of water potential is a motive force for water transport upstream in plants [14], much faster acropetal flow of xylem sap could be expected after local burning. Water drawing by gradient of water potential from living cells surrounding the xylem vessels could cause stem contraction. A second possible interpretation of stem contraction after local wounding is based on osmotic processes evoked by basipetal sap flow in xylem [3, 4, 15]. Rhodes et al. [16] showed that severe wounds (crushing or heat) induced flows in the xylem to other parts of the plant in a pattern determined by the vascular architecture. It was concluded that elicitors released by a severe wound were distributed systemically in the xylem. According to Malone’s concept, a solute-rich cell sap from the wounded tissue enters the xylem and travels via xylem in basipetal direction. Along the way, this solute-rich surge drains water from the healthy living cells surrounding the xylem via osmosis. Due to the dilution of the sap, this osmotic effect is less intensive with increasing distance from the wounded site and the surge is dampened [3, 4, 15].

The credibility of the first or second hypothesis could be verified by determining the direction of tobacco transpiration stream upon local burning, which is beyond the scope of this methodological paper and will be a subject of our further research. However at this point, both processes lead up to the same result—a loss of turgor in the living cells, which is followed by a decrease in their volume resulting in stem contraction. A decrease in volume of distant unwounded cells is supported by our previous results, where a delayed (about 5 min after burning) stomatal closure, followed by a decrease in transpiration, was detected in the tobacco leaf located below the burned leaf [17]. Moreover, tobacco xylem vessels are surrounded by a large number of cells (obvious from microscopic observations, data not shown) and the decrease in the volume of these cells could cause such an extensive stem narrowing observed in our case (Fig. 4).

The observed asymmetry in the stem narrowing on both stem margins (Fig. 4a) can be explained by different number of living cells between the affected xylem conduit and left or right stem margin. It is reasonable to assume that xylem conduit connected directly to the burned leaf will be most affected. Obviously, this vascular trace is close to the left margin of the stem. The number of cells between the affected xylem conduit and the left margin of the stem is much lower compared to number of cells between the conduit and the right margin. In other words, the water drainage from the area on the left is accomplished faster and thus the wound induced shift of the left stem margin culminates faster. It is believed that the higher number of cells between the affected xylem conduit and the right margin caused its delayed, but more pronounced shift. The return of the margins to their initial positions could be explained by the termination of water loss or the sap flow from the damaged site. As a result, the cells surrounding the conduit regain their original turgor.

## Conclusions

Our optical method allows non-invasive monitoring of both rapid and long-lasting deformations of opposite stem margins induced by the propagating hydraulic surge after local damage of a plant. The shift of both stem margins after local burning revealed a pronounced and asymmetric stem narrowing. The non-invasive measurement of these characteristics could be a helpful tool for the investigation of the mechanism of systemic response of plants to local damage.

## Additional files

**Additional file 1.** Description and algorithm of the software for position determination of the second dark fringe.

**Additional file 2.** Fresnel diffraction observed on partially transparent object.

**Additional file 3.** Burning process. Movie of burning of the 12^th^ leaf.
