# Supplementary material for: Non-invasive monitoring of hydraulic surge propagation in a wounded tobacco plant
Source: Plant Methods. 2018 May 25;14:38. doi: 10.1186/s13007-018-0307-6 (PMC5968581; doi:10.1186/s13007-018-0307-6)
Supplement: Supplementary file 1 — Additional file 1. Description and algorithm of the software for position determination of the second dark fringe. [file 13007_2018_307_MOESM1_ESM.pdf]

Description and algorithm of the software for position determination of the second dark fringe

The software tracks positions of the 2^{nd}DMin in least squares cubic splines fitted to the data from a row of a camera. A position of the minimum is determined by means of the second order derivative from the discrete data within an interval of specified width. The interval has to be narrow enough to only contain the 2^{nd}DMin and wide enough to cover the supposed shift of the 2^{nd}DMin between two consecutive images. The width of the interval was set to 100 pixels, which covered the stem margin shift by ±1 µm. The centre of the interval is at a position of the located 2^{nd}DMin. The starting position of the interval was set manually.

All the procedure of the 2^{nd}DMin determination in all images summarizes the following algorithm.

Initialization

- Set the length *L* (number of pixels) of a row in an image.
- Set the width *W* of an interval *I* in which the minimum is located (half of the width *W* has to be larger than the supposed shift of the 2^{nd}DMin between two consecutive records of camera).
- Set the approximate position *A* of the 2^{nd}DMin in image 1.
- Compute start i_L and end i_R indexes of the interval *I* whereas *A* is in the middle of the interval *I*.
- Set the total count *N* of images.

Determination of minima positions in images

- For image 1 to image *N* do
  - Read data from a row of an image.
  - Compute a least squares cubic spline for the data.
  - Compute values F_i of the fitted cubic spline at coordinates *i* of pixels of the row, where $i \in \langle 1, L \rangle$.
  - Compute the second order derivative from the discrete values F_i that indexes $i \in \langle i_L, i_R \rangle$.
  - Get an index *P* at which the second order derivative indicates the minimum.
  - Compute new start i_L and end i_R indexes of the interval *I* for locating the minimum in the next image whereas *P* is in the middle of the interval *I*.
