# Supplementary material for: Non-invasive monitoring of hydraulic surge propagation in a wounded tobacco plant
Source: Plant Methods. 2018 May 25;14:38. doi: 10.1186/s13007-018-0307-6 (PMC5968581; doi:10.1186/s13007-018-0307-6)
Supplement: Supplementary file 2 — Additional file 2. Fresnel diffraction observed on partially transparent object. [file 13007_2018_307_MOESM2_ESM.pdf]

Fresnel diffraction observed on partially transparent object

According to conclusions presented in Komrska (1967), the shape of intensity distributions (in diffraction patterns) of partially transparent objects remains unchanged except for its contrast and disturbances. The disturbances arise from the interference of light transmitted through the object and that diffracted by its edges. The visibility of disturbances is dependent on the difference in amplitudes of the interfering waves. The lower transparency of the object, the more interfering waves (transmitted and diffracted) differ in amplitude and disturbances diminish.

This supplementary material presents diffraction patterns from partially transparent objects, a solid glass rod, hollow glass rod and glass rod filled with green solutions of different transmittance.

In all cases, we used the same experimental set-up as in the paper (z_1 = 8 mm, z_2 = 297 mm, He-Ne laser, wavelength λ = 632.8 nm; for more details see the chapter „Methods" in the paper).

Solid glass rod

Parasitic fringes with high frequency are dominant in the pattern compared with fringes on the background that are crucial for our experiment and reflect the Fresnel diffraction on the margin of the rod. Parasitic fringes arise from the interference of light transmitted through the non-homogenous transparent solid glass rod and represent disturbances that would negatively affect determination of the required fringes position. The frequency of parasitic fringes is dependent on the width of the rod (Fig. 1, 2).

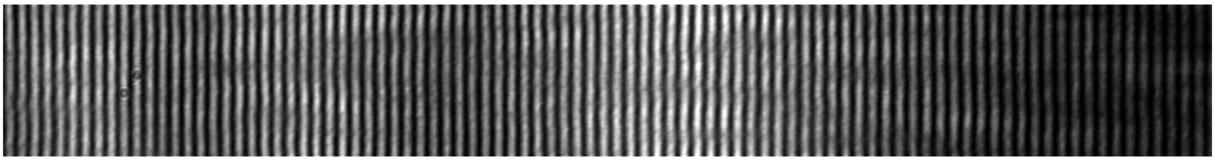

Fig. 1. A diffraction pattern observed on a solid glass rod with a diameter of 5 mm.

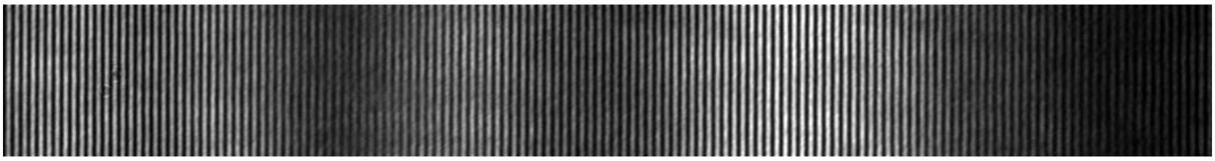

Fig. 2. A diffraction pattern observed on a solid glass rod with a diameter of 7.3 mm.

Empty hollow glass rod and rod filled with green solutions of different transmittance

A hollow glass rod was gradually filled with solutions of water and green acrylic dye with declining transmittance.

Parasitic fringes with high frequency are dominant in the pattern compared with fringes on the background that are crucial for our experiment and reflect the Fresnel diffraction on the external glass layer (margin) of the rod (Fig. 3). We distinguished two types of parasitic fringes, i.e. with higher and lower frequency (well visible in Fig. 4-6, in Fig. 3 are obvious after Fourier transform). The parasitic fringes arise from the interference of light transmitted through the hollow glass rod. Parasitic fringes represent disturbances that would negatively affect determination of the required fringes position. Other disturbances (like speckle pattern) appear when low concentration of dye solution was applied (Fig. 4). With increasing concentration of dye solution (gradually Figs. 4-5), the disturbances in diffraction pattern diminish and at the highest dye concentration the diffraction

pattern resembles that in opaque strip or the plant stem except the parasitic fringes (compare Fig. 6 and Fig. 1e in the paper). However, even if this diffraction pattern (Fig. 6) could possibly be applicable for the fringe position determination, the opaque strip or plant stem is more suitable for this experiment because of their non-transparency and thus an absence of parasitic fringes.

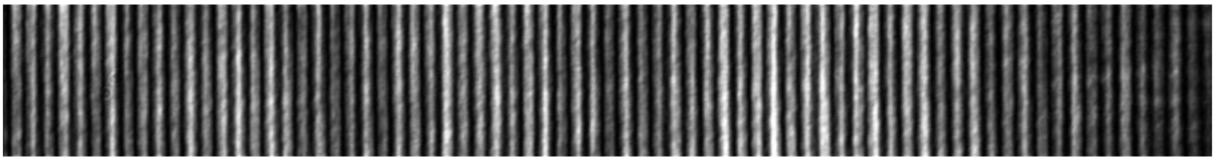

Fig. 3. A diffraction pattern observed on a hollow glass rod without green dye.

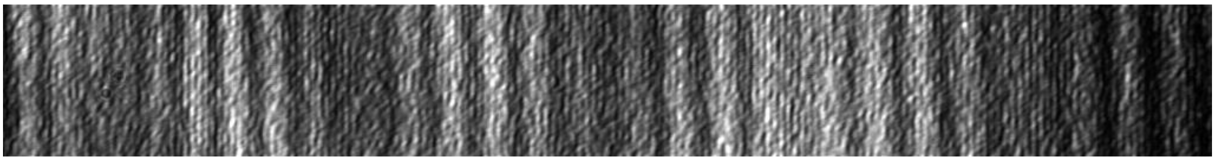

Fig. 4. A diffraction pattern observed on a hollow glass rod with the highest transmittance (0.19).

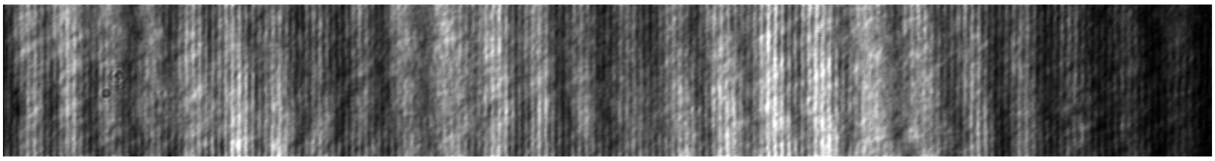

Fig. 5. A diffraction pattern observed on a hollow glass rod with a low value of transmittance (under detection limit).

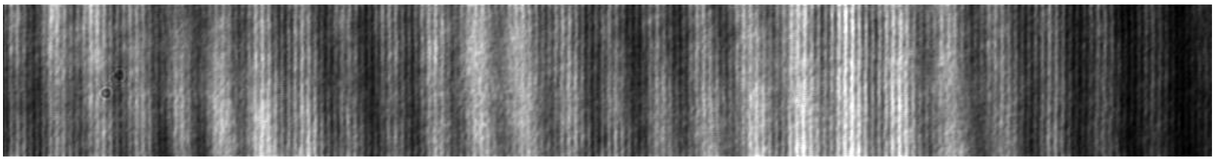

Fig. 6. A diffraction pattern observed on a hollow glass rod with the least value of transmittance (under detection limit).

References

1. Komrska J. Intensity and phase in Fresnel diffraction by a plane screen consisting of parallel strips. Optica Acta. 1967;14:127-46.
